# Transcriptome-wide based identification of miRs in congenital anomalies of the kidney and urinary tract (CAKUT) in children: the significant upregulation of tissue miR-144 expression

**DOI:** 10.1186/s12967-016-0955-0

**Published:** 2016-06-30

**Authors:** Ivan Jovanovic, Maja Zivkovic, Mirjana Kostic, Zoran Krstic, Tamara Djuric, Ivana Kolic, Dragan Alavantic, Aleksandra Stankovic

**Affiliations:** Laboratory for Radiobiology and Molecular Genetics, VINČA Institute of Nuclear Sciences, University of Belgrade, P.O. Box 522, 11001 Belgrade, Serbia; Nephrology and Urology Departments, University Children’s Hospital, Belgrade, Serbia; Medical Faculty, University of Belgrade, Belgrade, Serbia

**Keywords:** Biomarker, CAKUT, Development, Microarray, Integrative approach, Bioinformatics, MicroRNA, Pediatric

## Abstract

**Background:**

The genetic cause of most congenital anomalies of the kidney and urinary tract (CAKUT) cases remains unknown, therefore the novel approaches in searching for the common disease denominators are required. miRs regulate gene expression in humans and therefore have potentially therapeutic and biomarker properties. No studies thus far have attempted to explore the miRs in human CAKUT. We applied a new strategy to identify most specific miRs associated with CAKUT, in pediatric patients.

**Methods:**

Data from the whole genome expression, gathered from ureter tissue samples of 19 patients and 7 controls, were used for the bioinformatic prediction of miRs activity in CAKUT. We integrated microarray gene expression data and miR target predictions from multiple prediction algorithms using Co-inertia analysis (CIA) in conjunction with correspondence analysis and between group analysis, to produce a ranked list of miRs associated with CAKUT. The CIA included five different sequence based miR target prediction algorithms and the Co-expression Meta-analysis of miR Targets. For the experimental validation of expression of miRs identified by the CIA we used tissue from 36 CAKUT patients and 9 controls. The results of gene ontology (GO) analysis on co-expressed targets of miRs associated with CAKUT were used for the selection of putative biological processes relevant to CAKUT.

**Results:**

We identified 7 miRs with a potential role in CAKUT. The top ranked miRs from miRCos communities 4, 1 and 7 were chosen for experimental validation of expression in CAKUT tissue. The 5.7 fold increase of hsa-miR-144 expression in human tissue from CAKUT patients compared to controls (p = 0.005) was observed. From the GO we selected 7 biological processes that could contribute to CAKUT, which genes are potentially influenced by hsa-miR-144. The hsa-miR-200a, hsa-miR-183 and hsa-miR-375 weren’t differentially expressed in CAKUT.

**Conclusions:**

This study shows that integrative approach applied here was useful in identification of the miRs associated with CAKUT. The hsa-miR-144, first time identified in CAKUT, could be connected with biological processes crucial for normal development of kidney and urinary tract. Further functional analysis must follow to reveal the impact of hsa-miR-144 on CAKUT occurrence.

**Electronic supplementary material:**

The online version of this article (doi:10.1186/s12967-016-0955-0) contains supplementary material, which is available to authorized users.

## Background

Congenital anomalies of kidney and urinary tract (CAKUT) represent spectrum of developmental defects, which occur in approximately 1:500 liveborn children [1]. This very heterogeneous disease is comprised with different types of kidney and urinary tract disorders covering complete renal agenesis, different types of renal dysplasia, ureter malformations and obstructions of urine flow [2, 3]. CAKUT is also the most common cause of pediatric end-stage renal disease [1, 4]. The genetic and phenotypic heterogeneity, incomplete genetic penetrance and multifactorial nature of CAKUT creates difficulties in defining the genetic architecture of CAKUT [5]. Although the number of mutations in several genes, e.g. HNF1B, PAX2, DSTYK have been identified in syndromic CAKUT, familiar forms or isolated patients, they were detected in no more than 10 % of patients and show no uniform genetic pattern [6, 7]. The contribution of the most of previously implicated genes was shown to be much smaller than expected [8]. The genetic cause of most CAKUT cases remains unknown, therefore the novel approaches in searching for the common disease denominators are required.

MicroRNAs (miRs) are small, non-coding RNAs with potentially therapeutic and biomarker properties that regulate gene expression, either by promoting the degradation of mRNA or by inhibiting the translation [9]. The degradation of miR target represents the predominant mode of action in mammalian organisms [10]. It was described that conditional loss of kidney miRs induced by deletion of Dicer leads to formation of CAKUT in mice [11]. Although these findings imply the important role of miRs in the development of kidney and urinary tract, the information about specific miRs that contribute to CAKUT are scarce and largely deduced from non-CAKUT contexts [12]. No studies thus far have attempted to explore the miRs in human CAKUT.

As the number of described miRs grows every day and by taking into account that different miRs could share the same target or that single miR could target multiple mRNAs, the experimental investigation of all possible interactions becomes very difficult. Therefore, bioinformatic algorithms represent valuable tools for prediction of target sites in the mRNA 3′UTRs for every miR. Although the sequence based types of these tools differ, they all rely on the established set of canonical rules for miR—target mRNA pairing. These rules represent sequence complementarity of the miR seed region and the 3′UTR of target mRNA, and the evolutionary cross species conservation [13]. Coexpression meta-analysis of miR target genes (CoMeTa) is more comprehensive miR target prediction approach. It uses sequence based prediction just to seed the procedure and performs further target detection by relying exclusively on the mRNA transcriptome data sets available in public databases. By creating the co-expression lists of putative targets, CoMeTa efficiently ranks higher the true positive miR target predictions. The coexpression analysis was also used for the identification of miR communities involved in the synergistic modulation of cohorts of genes that regulate similar processes and for the analysis of biological functions of coexpressed miR target clusters [14].

We applied a new strategy to identify miRs associated with CAKUT by employing the multivariate statistical technique, Co-inertia analysis (CIA) in conjunction with correspondence analysis and between group analysis (BGA) [15, 16]. The method was previously successfully applied for confirmation of known miRs and determination of new miRs with significant biological effect on certain disease development and outcome [15–18]. The aim of the study was to identify the most specific miRs in CAKUT, by employing the CIA, which integrates microarray gene expression data and miR target predictions from multiple prediction algorithms (sequence based and CoMeTa). The second aim was to identify if certain resulting miRs share a significant proportion of target genes and downstream regulatory networks, and to use this layout to select miRs for experimental validation of miRs expression in ureter tissue from children with CAKUT.

## Methods

### Patients and controls

The total of 55 patients (19 patients for the microarray gene expression experiment/36 patients for the mature miRs qPCR expression validation experiment) with diagnosis of CAKUT, consecutively admitted to the University Children’s Hospital, Departments of Nephrology and Urology, Belgrade, Serbia, from 2011 to 2016, who were selected for ureter corrective surgery were included in the study. Inclusion criterion was that none of the family members were affected by CAKUT. Diagnosis was based on ultrasonography before and after birth, and confirmed by IVU and radioisotope renography (^99m^Tc-DTPA). Reflux was diagnosed on voiding cysto-urethrography (VCUG). The patients presented with one or more of the following CAKUT conditions: ureteropelvic junction (UPJ) obstruction, vesicoureteric junction (VUJ) obstruction, primary obstructive megaureter (OMU) and primary vesicoureteral reflux (VUR). The primary nature of OMU and VUR was confirmed by excluding secondary causes, such as neurogenic and non-neurogenic voiding dysfunction, or bladder outlet obstruction (e.g. posterior urethral valves). The age of patients ranged from 0.1 to 6 years. A complete patient’s and family medical history and demographic data were obtained. Sixteen controls (7 for the microarray gene expression experiment/9 for the mature miRs expression validation experiment by qPCR) were recruited consecutively among healthy adult kidney donors. Ureter tissue used as control tissue collected during the procedure of kidney transplantation at the University Children’s Hospital, Department of Nephrology, Belgrade, Serbia during the 5 year period (2011–2016).

All participants in this study were Caucasians of Serbian origin. The Ethical University Research Comity approved the study. Adult participants and children’s parents gave written informed consent.

### Procurement of tissue specimens and total RNA isolation

The ureter segment samples were obtained with minimal manipulation, immediately after performing surgical interventions using standard techniques. All tissue samples were stored in RNAlater solution at the temperature of −20 °C until the isolation of total RNA.

The equal sizes of intact ureters were sliced transversely in sterile conditions to make the samples as comparable as possible. Sliced ureter tissue was homogenized in liquid nitrogen. Total RNA was extracted by TRI Reagent^®^ Solution (Ambion) according to the manufacturer’s instructions. Structural integrity and concentration of total RNA and cRNA were determined using Agilent RNA 6000 Nano Kit on Agilent 2100 Bioanalyzer.

### Whole genome gene expression

All samples included in whole genome gene expression experiment had an RNA integrity number >7. Linear RNA amplification procedure and synthesis of biotin labeled cRNA was performed with 200 ng of total RNA using TargetAmp™-Nano Labeling Kit for Illumina^®^ Expression BeadChip^®^ (Epicentre). The RNeasy MinElute Cleanup Kit (QIAGEN) was used for purification of synthesized cRNA.

Total amount of 750 ng of biotin labeled cRNA was used for hybridization on each array of HumanHT-12 v4 Expression BeadChip (Illumina). Using this high-throughput technology, it was possible to determine expression profiles of more than 47,000 different, well-characterized transcripts. Hybridization procedure was conducted according to Illumina^®^ Direct Hybridization Assay protocol. Hybridized BeadChips were scanned with iScan microarray scanner (Illumina). Bead intensity data were background subtracted and quantile normalized using Gene Expression v1.9 module of GenomeStudio software 2011.1 (Illumina).

### miR target prediction

For initial Co-inertia analysis (CIA), the five sequence based miR target prediction algorithms were used: TargetScan (v4.1) and TargetScanS (v4.1) [19, 20], PicTar4way and Pictar5way [21], and miRanda [22] according to Madden et al. [16]. Each of these sequence based prediction algorithms utilizes the complementarity of the miR seed and the cross species conservation in their predictions. The miR target prediction data for CIA input, extracted from these databases, was organized in gene/miR frequency tables of counts of predicted targets per gene for each of the algorithms. The gene/miR frequency tables for sequence based predictions were provided by the authors [16]. Additionally, we have collected the list of targets for each miR present in the first 50th percentile of CoMeTa lists from publicly available database [14] and created gene/miR frequency table for the additional CIA performed on the same microarray gene expression data set.

### Co-inertia analysis

The Co-inertia analysis was performed on the raw data of whole genome mRNA expression results from 7 control and 19 CAKUT human ureter tissue samples. This multivariate data integration technique uses the entire gene expression dataset, and therefore does not require any prefiltering of the transcriptomic data. The CIA was used to combine 2 linked datasets (gene expression data and predicted miR target information) for the same genes. The unsupervised CIA was first applied for visualization and exploration of gene expression signatures in different samples and how these differences related to the occurrence of miR target sites. Supervised analysis was then conducted using Between Groups Analysis (BGA) for the identification of miRs associated with CAKUT. The output from supervised CIA performed on sequence based predictions were five rank lists of miRs associated with the disease, 1 for each of the 5 miR target prediction algorithms. Lists were combined and CIA average ranking was calculated using consistency among the methods, according to previous study [16].

In order to determine the most specific miRs with the greatest impact on CAKUT development, we performed the additional supervised CIA on CoMeTa miR target predictions [14]. CoMeTa was based on the experimental data, and therefore was suitable for additional correction of CIA results from sequence based prediction algorithms. List of miRs selected from supervised CIA performed on sequence based predictions was intersected with the top 15 % of miRs from the CIA performed on CoMeTa predictions for extraction of the most specific miRs predicted to be associated with CAKUT (Additional file 1). The complete analysis was performed by the MADE4 R package [23], based on dataset analysis script [16], which we have modified for the operation with Illumina platform.

In order to facilitate the selection of miRs for experimental validation, we have additionally analyzed the final list of miRs associated with CAKUT for the presence of different miR communities. These communities are groups of miRs that share a significant proportion of target genes and downstream regulatory networks, as revealed by co-expression analysis [14].

We have examined the community layout of miRs associated with CAKUT, and according to the CIA average ranking, we selected the best ranked miR from each of the communities for the experimental validation.

### Mature miRs expression validation by quantitative real-time PCR

The validation experiment for mature miRs expression in CAKUT included two steps: generation of cDNA from total RNA followed by TaqMan^®^ Real-time PCR. Total RNA was reverse transcribed, using TaqMan^®^ miR Reverse Transcription Kit following the manufacturer’s protocol (Applied Biosystems, Inc., Foster City, CA). TaqMan^®^ miR assays included specific RT Primers and TaqMan^®^ Probes to quantify mature forms of miR chosen for experimental validation. Assays used in this study were: hsa-miR-200a (ID 000502), hsa-miR-183 (ID 002269), hsa-miR-375 (ID 000564), hsa-miR-144 (ID 002676). Stably expressed reference RNU44 (ID 001094) was used for normalization. All reactions were performed in duplicates in a 96-well plate at 95 °C for 10 min followed by 40 cycles of 95 °C for 15 s followed by 60 °C for 1 min in ABI 7500 system (Applied Biosystems, Inc., Foster City, CA). No-template controls were included in both reverse transcription and qPCR procedures. A threshold cycle (CT) was obtained in the exponential phase of amplification.

Statistical analysis of miR relative expression was performed on 2^−ΔCt^ values of CAKUT and control ureter samples [24]. DataAssist™ Software v3.01 (Applied Biosystems, 2012) was used for handling of ABI 7500 system (Applied Biosystems, Inc., Foster City, CA) exported experiment design files, for calculation of 2^–ΔCt^ values and estimating relative quantification [24]. The SPSS Statistics v20 software (IBM) was used for the analysis of the differences in relative miR expression levels between CAKUT and control group using Mann–Whitney U test, which is suitable for comparison of two unpaired groups of continuous variables that do not follow a normal distribution. Values of p <0.05 were considered statistically significant.

### Selection of gene ontology functional categories of statistically significant differentially expressed miRs

The CO-operational level (COOL) analysis was previously developed for the clustering of predicted miR targets that showed the highest extent of co-expression [14]. These clustered subsets of miR targets were used for the gene ontology (GO) analyses in order to find the most significant GO functional categories associated with clustered genes [14]. We used these publicly available data to select the biological processes relevant to CAKUT pathology, which are controlled by significant miR from our study. The categories were selected according to biological meaning and the rule that GO analysis p values with Bonferroni adjustment must be ≤0.05.

## Results

### Co inertia analysis results

**Fig. 1:**
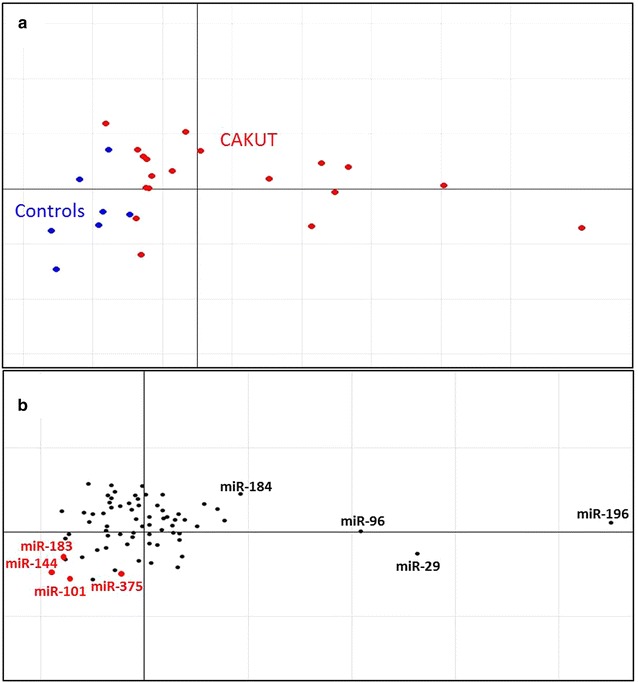
Example of the unsupervised CIA. The *plot* shows the axes of the unsupervised CIA performed on the whole genome gene expression data of CAKUT patients and controls. The gene/miR frequency table generated with TargetScanS was used to make this figure. **a** Shows the projection of CAKUT and control samples. **b** Shows the projection of the miRs. Motifs in the opposite orientation relative to the origin are associated with that group of samples. Therefore, by the observation of the *plots*, miRs hsa-miR-144, hsa-miR-101, hsa-miR-183 and hsa-miR-375 are potentially associated with CAKUT according to their position

A combination of unsupervised CIA (using correspondence analysis) and supervised CIA (using BGA) was employed to simultaneously analyze mRNA expression levels from microarray and miR target prediction information in the 3′UTRs of the same genes. Five such analyses were conducted using sequence based prediction programs. Additionally, the CIA was performed using target predictions from CoMeTa for further filtering of the results acquired from CIA done using sequence based predictions. Figure 1 shows an example of unsupervised analysis of CIA using TargetScanS target prediction program. The plot is in 2 parts where Fig. 1a depicts a correspondence analysis plot of CAKUT and control samples and Fig. 1b depicts miRs associated with these samples. The 2 plots are interrelated; samples in part (A) are associated with miRs in part (B) in the opposite orientation relative to the origin. In Fig. 1a, we can see the split in the data along the horizontal axis, separating the controls and CAKUT. In Fig. 1b, four miRs finally selected for experimental validation (hsa-miR-144, hsa-miR-101, hsa-miR-183 and hsa-miR-375) were in the opposite direction from the origin compared to CAKUT samples in Fig. 1a suggesting that these miRs might be responsible for the downregulation in the CAKUT gene expression data.

To systematically identify miRs specifically associated with CAKUT, a supervised analysis was conducted by combining CIA and BGA. By applying the previously described consistency method [16] for the selection of miRs associated with CAKUT we got 18 miRs that appeared in at least 2 sequence based prediction algorithms (Additional file 2). The resulting miRs were used for the intersection with the top ranked miRs of the CIA performed on CoMeTa miR target predictions. In that way, a fraction of false positive results was filtered out. We received a notably reduced list containing 7 miRs (hsa-miR-144, hsa-miR-101, hsa-miR-375, hsa-miR-200a, hsa-miR-183, hsa-miR-495, hsa-miR-222) with a potential role in CAKUT development. All of the miRs, except hsa-miR-222, appeared in pairs, each belonging to different miR communities and shared the significant proportion of target genes revealed by co-expression analysis. These were community 4, 7 and 1 according to the miRCos procedure [14].

### Experimental validation of miRs expression in CAKUT

According to the CIA average ranking, we have chosen the better ranked miRs from communities 4 and 1 (hsa-miR-144 and hsa-miR-183 respectively) for experimental validation in this study (Table 1). Both miRs from community 7 (hsa-miR-375 and hsa-miR-200a) had high rankings in the CIA (Table 1), thus both were experimentally validated as well. As the annotation about the 5′/3′ hairpin precursor predominantly wasn’t present in the results of CIA we have chosen to validate the mature miRs dominantly abundant in human cells (the sequence without * in the annotation) [25]. The chosen mature forms for validation were: hsa-miR-144-3p, hsa-miR-200a-3p, hsa-miR-375-3p and hsa-miR-183-5p. The annotations in further text will be without strand information.

**Table 1:** The most specific miRs predicted to be associated with CAKUT

miRs from intersection	mirCos community	CIA average ranking
*hsa-miR-144*	4	*7*
hsa-miR-101	4	10.5
*hsa-miR-375*	7	*4.3*
*hsa-miR-200a*	7	*2*
*hsa-miR-183*	1	*11*
hsa-miR-495	1	12
hsa-miR-222	70	6.5

The final list of miRs present both in CIA performed on sequence based miR target predictions and CIA performed on CoMeTa predictions. miRs from the same community that have higher average ranking were chosen for experimental validation (italics)

**Fig. 2:**
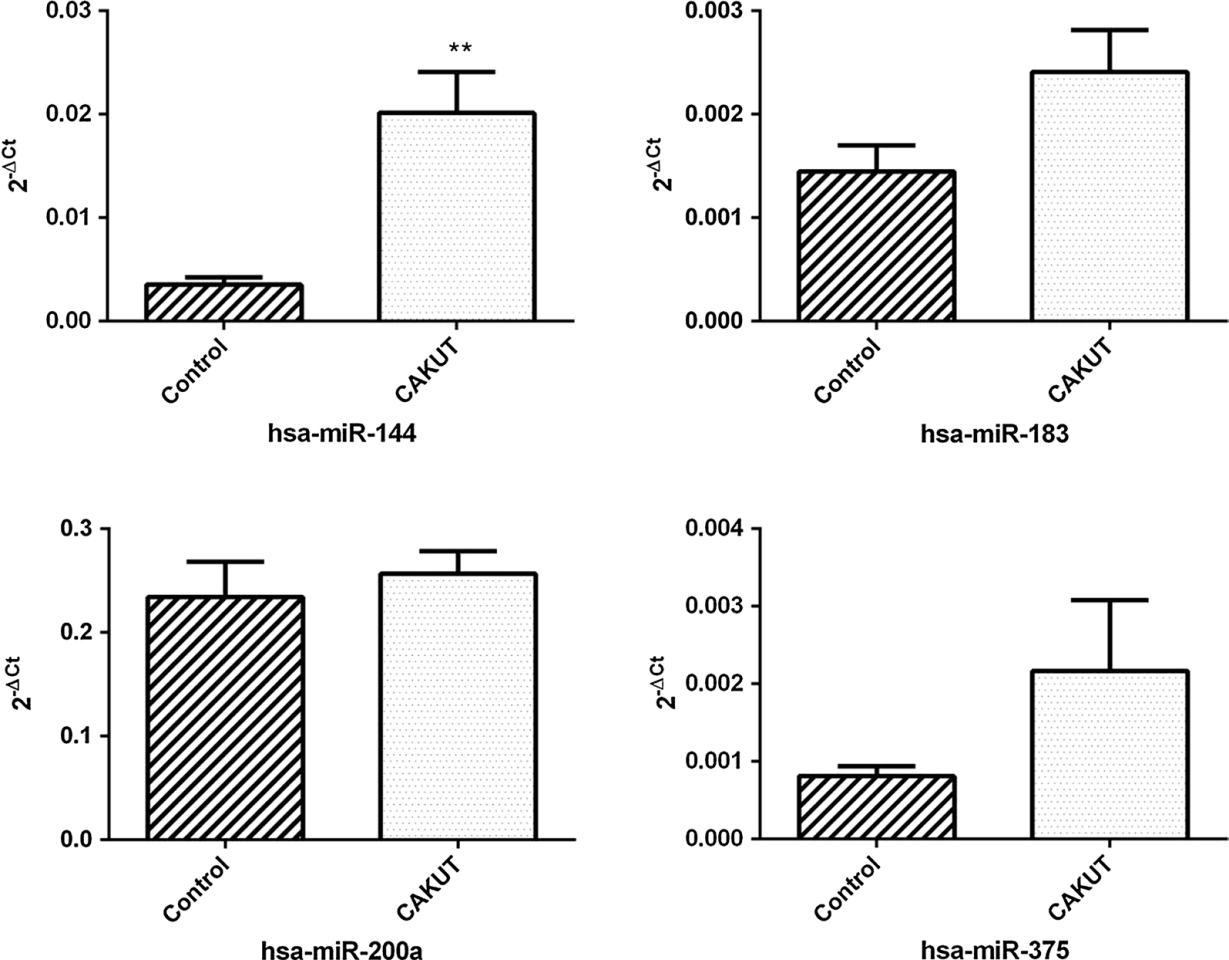
Difference in relative expression of hsa-miR-144, hsa-miR-183, hsa-miR-200a, and hsa-miR-375 between CAKUT patients (n = 36) and controls (n = 9). Relative miR levels were standardized against RNU44 endogenous control and presented as mean 2^−ΔCt^ values ±SEM. Mann–Whitney U test was used to compare two unpaired groups of continuous variables that do not follow a normal distribution, **p ≤ 0.01

The present study showed a statistically significant 5.7 fold increase of hsa-miR-144 expression in human ureter tissue from CAKUT patients (n = 36) compared to control ureter tissue (n = 9) (p < 0.01) (Fig. 2; Table 2). Mann–Whitney U test was used to compare two unpaired groups of continuous variables that do not follow a normal distribution. The hsa-miR-200a, hsa-miR-183 and hsa-miR-375 were expressed in both patient’s and control’s tissue, but statistically significant difference in relative expression was not observed (Fig. 2; Table 2).

**Table 2:** Differential expression of miRs in CAKUT group compared with controls

micro RNA	Fold change	Man–Whitney U p value (two tailed)
hsa-miR-144	5.7	0.005
hsa-miR-200a	1.1	0.7
hsa-miR-183	1.7	0.3
hsa-miR-375	2.7	0.9

Fold change was calculated using 2^−ΔCt^ values of relative miR levels standardized against RNU44 endogenous control

### Assigning the CAKUT related biological functions to hsa-miR-144

**Fig. 3:**
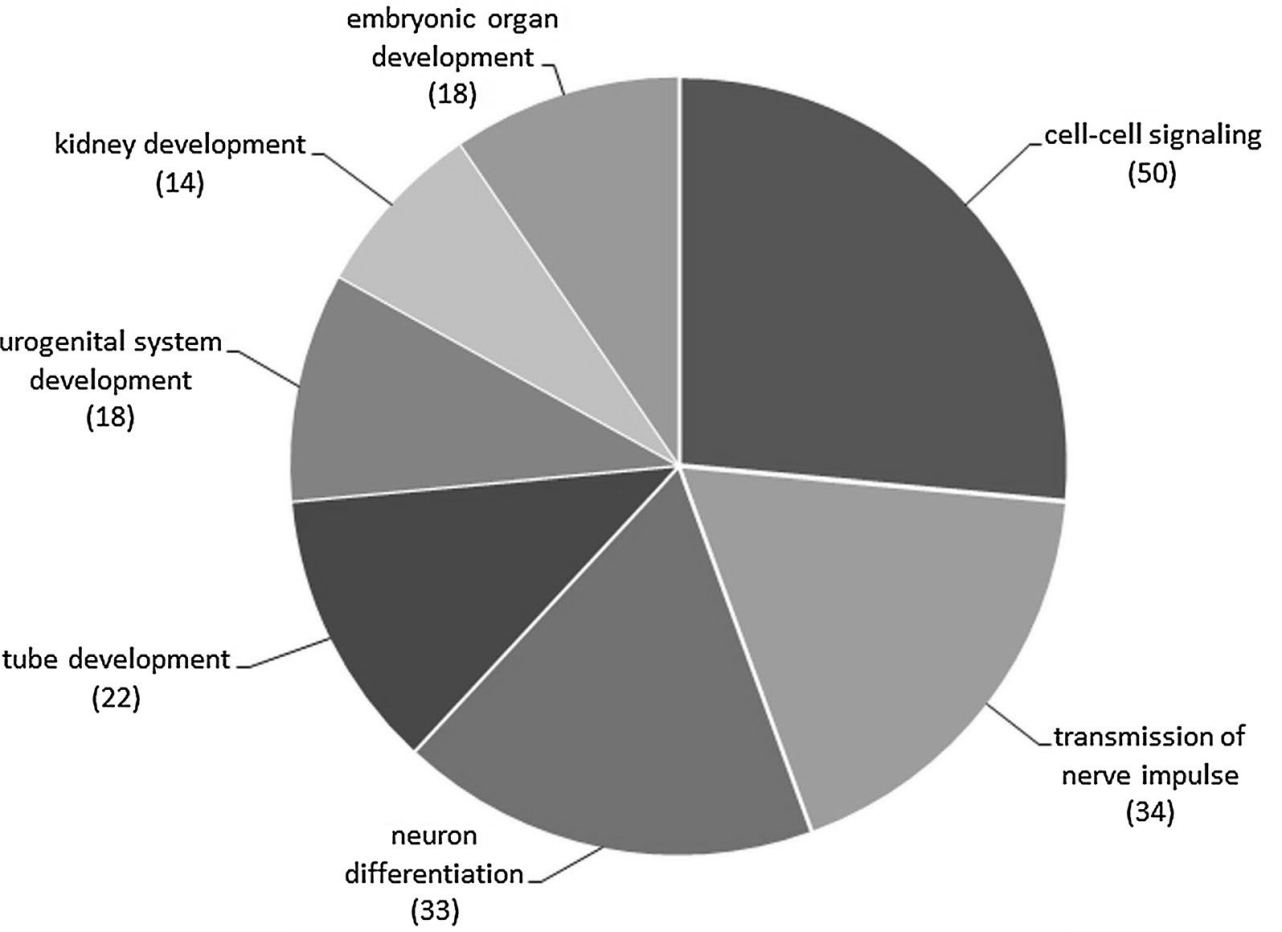
CoMeTa predicted hsa-miR-144 target genes contributing to biological processes involved in the development of CAKUT. The *chart* shows the relative ratio of the number of genes associated with different functional GO categories (biological processes). Biological process categories were selected according to biological meaning and the rule that GO analysis p values with Bonferroni adjustment must be ≤0.05. The number of CoMeTa predicted target genes of hsa-miR-144 associated with each category is shown in parentheses

Gene targets of the hsa-miR-144 predicted by the co-expression method of the CoMeTa were analyzed by Gennarino et al., using COOL analysis to produce gene clusters that showed the highest extent of co-expression [14]. The two gene clusters with high reciprocal expression relationships, containing 1024 and 511 genes, resulted from the COOL performed on hsa-miR-144 predicted targets. These clusters were subjected to GO analysis and it was found that gene cluster containing 511 genes showed enriched categories of biological processes potentially related to CAKUT. The authors got 29 statistically significant GO terms after the multiple testing corrections [14] (Additional file 3). We performed the selection of the most important GO functional categories that describe biological processes responsible for normal development of kidney and urinary tract according to the contemporary literature information. Seven GO terms were selected and presented as a pie chart showing the number of predicted target genes of hsa-miR-144 (Fig. 3). Of the selected biological processes, cell–cell signaling process contained the largest number of hsa-miR-144 predicted targets (50 target genes). The neural factors were described to be important in urinary tract development [26], thus we have selected the transmission of nerve impulse and neuron differentiation as important processes controlled through hsa-miR-144 targeting of the notable number of genes (34 and 33 respectively). As for the developmental processes, there were four GO terms describing the potential of direct participation of hsa-miR-144 in CAKUT. These were tube development (22 target genes), embryonic organ development (18 target genes) urogenital system development (18 target genes) and kidney development (14 target genes) (Fig. 3).

## Discussion

The genetic cause of most CAKUT cases remains unknown. Urogenital tract and renal system phenotype depends on genetic, epigenetic and environmental factor interactions [27, 28]. It was shown that miRs could modulate genomic stability and mismatch repair [29]. More than 30 % of the human genes could be regulated by miRs [30] and miRs could represent biomarkers for environmental exposure [31]. In this study we applied the comprehensive bioinformatical approach in identification of miRs associated with human CAKUT. Predicted miRs were experimentally validated to be expressed in ureter tissue: hsa-miR-144, hsa-miR-375, hsa-miR-183 and hsa-miR-200a. We revealed a 5.7-fold induction of hsa-miR-144 in CAKUT samples compared to controls. Although it was suggested that elevated levels of hsa-miR-144 in body fluids could represent a biomarker of diabetic kidney injuries [32], the role and biomarker potential of this miR in CAKUT was not investigated.

The hsa-miR-144 gene is located in a polymorphic region (17q11.2) rich with CNVs, according to the database of genomic variants [33]. CNVs are functional variants which can alter the miR expression through the shift in miR gene dosage or the positioning of the miR gene regulatory elements [34]. Recent studies have investigated the association of CNVs with CAKUT and revealed that a significant proportion of patients harbor submicroscopic chromosomal imbalances, among others, in the 17q11-12 region [35, 36]. Therefore, hsa-miR-144 might be upregulated by specific CNV genotype. Long-range interactions between DNA segments affected by CNVs might also directly modify the miR expression pattern [34]. However, the mechanism responsible for miR expression regulation should not be interpreted solely through genetic variation due to the complexity of other epigenetical and environmental interactions [5, 37]. The inverse correlation pattern was described between hsa-miR-144 expression and genome-wide methylation status in cancer [38]. Epigenetic modifications have been postulated as a mechanism that facilitates the interaction between environmental factors and the genome during development, and its impact on disease susceptibility. As epigenetic modifications are reversible and susceptible to environmental stress (such as in utero environment), thus enabling developmental and temporal variability in gene expression patterns [5], these modifications could also lead to dysregulation of miR expression in early development, that could even remain postnatally. In mammals, multigenerational environmental effects have been documented by either epidemiological studies in human or animal experiments in rodents [39]. Steady state cellular miR levels represent the balance between miR biogenesis, influenced with described CNV and epigenetic mechanisms, and turnover [40]. However, the different turnover kinetics between certain miR isoforms and between different miRs is still not fully explained [40]. Therefore, future studies should also take into consideration, besides biogenesis, the turnover mechanisms and research of yet unknown molecular chaperones responsible for this process.

The development of the kidney and urinary tract is a complex process involving precise temporal and spatial modulation of gene expression responsible for correct proliferation, differentiation, and morphogenesis [41]. Analysis of the coexpression clusters of CoMeTa predicted hsa-miR-144 targets from the Co-Operational Level (COOL) analysis, and GO analysis of these clusters [14], revealed that a cluster of 511 putative targets was enriched with describes biological processes relevant for CAKUT pathology (Fig. 3). Certain GO biological processes enriched in the cluster (kidney development, urogenital system development, tube development, embryonic organ development) suggest that deregulated expression of hsa-miR-144 might contribute to impaired development of kidney and urinary tract. Although in this study we could not investigate the expression of hsa-miR-144 in human tissue during development, but after birth, certain developmental factors which represent crucial controllers of developmental stages could have prolonged activity in later stages after birth in CAKUT etiology. For example, TGF-β1, one of the main factors responsible for the kidney development, shows upregulation in human obstructive renal dysplasia [42] and prolonged expression in stenotic tissue of patients with UPJ obstruction [43]. In contrast with dysplastic tissues, normal prenatal and postnatal kidneys showed no significant TGF-β1 expression [42]. These data support the possibility of prolonged activity of certain miRs as important epigenetic factors in different stages of urogenital system development, where dysregulation of these miRs could lead to destabilization of important signaling pathways and to result in congenital malformations.

One of the main limitations of this study is the age difference between CAKUT patients and controls. However, miR-144 was found to be the sole miR that was consistently upregulated in the aging human and nonhuman primate cerebellum and in nonhuman primate cortex [44]. It was upregulated in the cerebromicrovascular endothelial cells of aged rats compared to young rats [45] and in skeletal muscle of old rhesus monkeys compared to young rhesus monkeys [46]. Although this highly conserved miR shows age dependent upregulation, this should not bias the results of our study in which hsa-miR-144 was upregulated in ureter samples of children with CAKUT compared to adult controls. Nevertheless, it is of importance to validate our results in age matched ureter tissue samples. Another limitation is the omitted kidney tissue. However, we deliberately selected the uniform tissue for expression analysis to improve the power of the study. Since the most common kind of surgical treatment in children with CAKUT is corrective surgery of the urinary tract, the human ureter tissue was selected as a target tissue for examination in this study. Ureter tissue has the same developmental origin as collecting duct system of the kidney, major and minor calyces and the renal pelvis [47], and the epithelium of the adult kidney consists of a number of specialized cell lineages where some originate from branching epithelial cells of the ureteric bud [41]. A drawback of our approach is that it assumes that the change in gene expression signature is solely the result of miR activity. It overlooks the activity of other transcription regulation factors. Another drawback is that the association of the miRs with CAKUT is based on miR target prediction. These could be the reasons for hsa-miR-183, hsa-miR-200a and hsa-miR-375 expression deviations from results of the CIA. However, the data about experimentally confirmed miR-mRNA interactions are still very scarce, thus the predictions still represents the most comprehensive approach. Although we used the advanced novel approach for filtering the prediction data, these results still need comprehensive experimental validation. Also, the discrepancy in the microarray platforms in our study and the one used for CoMeTa [14] might lead to overseeing the certain genes by the CIA based on CoMeTa miR target predictions, and thus influence the detection of miRs associated with CAKUT.

## Conclusions

CAKUT is the most common cause of pediatric end-stage renal disease. Therefore, the discovery of novel biomarkers and therapeutic targets, applicable in future prevention and therapy, is of great importance. In order to find key regulatory agents associated with CAKUT pathology, the complexity of the responsible molecular processes could only be dissected using integrative approaches and systems biology. Differentially regulated miRs may represent innovative biomarkers for diagnosis and prognosis of the disease. This was the first ex vivo human study that investigated the expression of miRs: hsa-miR-144, hsa-miR-200a, hsa-miR-375 and hsa-miR-183 in human CAKUT. The results of upregulated expression of hsa-miR-144 should represent the guide mark for further genetical and functional studies on mechanisms of miR expression and activity, involved in the development of urinary tract anomalies.

## Additional files

10.1186/s12967-016-0955-0 The starting point for every performed analysis are two tables: one table of gene expression values for G genes from N samples (N microarrays) and the other table giving the counts of predicted target sites for M miRs in the same G genes. There were 5 tables of predicted miR target counts from 5 sequence based prediction algorithms and 1 table of miR target predictions from CoMeTa. Therefore, 6 analyses were performed on the same gene expression data. These tables GxM and gene expression tables GxN were analyzed using non symmetric correspondence analysis (NSC) and linked using CIA from made4 R package. This is unsupervised method, used for data exploration and visualization purposes. The supervised analysis was performed by applying between group analysis (BGA), which takes class information and a NSC analysis and finds axes or vectors that best discriminate preassigned groups by maximizing the between group variance. This technique was used to automate the analysis by specifying a predetermined split in the microarray samples (CAKUT and control samples), and to identify putative regulating miRs associated with the split. The result of a BGA analysis on 2 groups is a ranked list of miRs associated with CAKUT, for every prediction algorithm used. The miRs which ranked highly (in the top 20) with two or more of the 5 sequence based programs were used for the intersection with the miRs which ranked in top 15 % with CoMeTa predictions.
10.1186/s12967-016-0955-0 Top ranked miRs in Co-inertia analysis (CIA) performed with five different miR target prediction algorithms.
10.1186/s12967-016-0955-0 Statistically significant GO terms from Gene Ontology analysis performed on a cluster of 511 hsa-miR-144 target genes.

## Abbreviations

miR: micro RNA
CAKUT: congenital anomalies of the kidney and urinary tract
CIA: co-inertia analysis
GO: gene ontology
BGA: between group analysis
COOL: CO-operational level analysis
3′UTR: 3′ untranslated region
CoMeTa: coexpression meta-analysis of miR target genes
VCUG: voiding cysto-urethrography
UPJ: ureteropelvic junction
VUJ: vesicoureteric junction
OMU: primary obstructive megaureter
VUR: primary vesicoureteral reflux
CNV: copy number variations
