# Supplementary material for: Transcriptome-wide based identification of miRs in congenital anomalies of the kidney and urinary tract (CAKUT) in children: the significant upregulation of tissue miR-144 expression
Source: J Transl Med. 2016 Jun 30;14:193. doi: 10.1186/s12967-016-0955-0 (PMC4929761; doi:10.1186/s12967-016-0955-0)
Supplement: Supplementary file 2 — 10.1186/s12967-016-0955-0 Top ranked miRs in Co-inertia analysis (CIA) performed with five different miR target prediction algorithms. [file 12967_2016_955_MOESM2_ESM.pdf]

Supplementary table 1. Top ranked miRs in Co-inertia analysis (CIA) performed with five different miR target prediction algorithms.

miRanda	TargetScan	TargetScanS	Pictar4way	Pictar5way	miRs associated with CAKUT
miR-194	**miR-191**	**miR-139**	miR-369-5p	**miR-200a**	
**miR-144**	miR-423	**miR-375**	**miR-191**	**miR-222**	
miR-577	**miR-210**	**miR-101**	**miR-200a**	miR-302c-star	**miR-144**
miR-802	miR-134	**miR-183**	miR-149	miR-205	**miR-152**
miR-616	**miR-375**	**miR-144**	**miR-215**	miR-143	**miR-210**
miR-376a	miR-30-3p	**miR-1**	**miR-375**	**miR-210**	**miR-375**
miR-302c	miR-409-3p	**miR-206**	**miR-148a**	miR-221	**miR-1**
miR-585	miR-431	miR-145	miR-16	**miR-148b**	**miR-101**
**miR-338**	miR-224	miR-106	**miR-148b**	miR-21	**miR-139**
miR-493-5p	**miR-139**	miR-17	**miR-210**	**miR-148a**	**miR-148**
miR-30c	**miR-495**	miR-20	**miR-222**	**miR-206**	**miR-148a**
miR-379	miR-299-5p	miR-181	miR-23b	**miR-338**	**miR-148b**
**miR-495**	**miR-148**	miR-23	miR-23a	miR-199a-star	**miR-183**
miR-569	**miR-152**	**miR-148**	miR-340	**miR-144**	**miR-191**
miR-607	miR-100	**miR-152**	miR-15b	**miR-1**	**miR-200a**
miR-520h	miR-99	miR-199	miR-136	miR-155	**miR-206**
miR-520d	miR-433-5p	miR-141	miR-195	**miR-152**	**miR-215**
miR-658	**miR-183**	miR-192	**miR-101**	miR-19b	**miR-222**
miR-200b	miR-485-5p	**miR-215**	miR-15a	miR-19a	**miR-338**
miR-450	miR-486	miR-10	miR-181b	miR-142-5p	**miR-495**

miRs associated with CAKUT appeared in at least two prediction algorithms are highlighted in bold
